# The Influence of Chinese Machiavellianism and Moral Identity on the Level of Anxiety in Moral Dilemma Situations in Chinese Students

**DOI:** 10.3389/fpsyg.2021.615835

**Published:** 2021-02-25

**Authors:** Shujun Tang, Kai Li

**Affiliations:** ^1^School of Marxism, Jiangxi Normal University, Nanchang, China; ^2^School of Psychology, Jiangxi Normal University, Nanchang, China

**Keywords:** Machiavellianism, ethical values, anxiety, moral dilemma, psychological stress

## Abstract

Based on the conflict-of-values theory, this study examines the influences of Machiavellianism and ethical values on anxiety in college students when they face moral dilemmas. Questionnaires on the Chinese equivalent of Machiavellianism, moral identity, and anxiety were completed by 115 Chinese college students. The results suggest that Machiavellianism and ethical values influence anxiety, and the interaction between ethical values and Machiavellianism is significant—among individuals with high ethical values, those with high levels of Machiavellianism exhibit markedly higher levels of anxiety than those with low levels of Machiavellianism. However, among individuals with low ethical values, there is no difference in anxiety between those with low or high levels of Machiavellianism. This research, depicting complex relationships among anxiety, Machiavellianism, and ethical values, suggests that leading people to do good deeds not only requires high ethical values but also necessitates the resistance to negative values such as Machiavellianism.

## Introduction

Human behavior is influenced by individuals' values. To realize the Chinese dream of national rejuvenation, the Chinese government advocated 24 core socialist values, including prosperity, democracy, civility, harmony, freedom, equality, justice, the rule of law, patriotism, dedication, integrity, and friendliness (Wu, 2009). These values are intended to guide people to do good things. This doctrine has been used to develop a way of thinking for students.

Human beings are selfish, dishonest, and sometimes evil (Locke, 1967). According to Schwartz's (1994) circumplex model of values, the presence of two competing values would cause psychological stress. The core social values pool positive energy, which is contrary to selfish and evil values. For example, core social values encourage people to be patriotic, dedicated, integrous, and genial, while people who are high in Machiavellianism are likely to engage in unethical and counterproductive work behaviors (Winter et al., 2004).

Given that contemporary college students are influenced by core social values, what psychological and behavioral responses do they have when they face moral dilemmas? The purpose of this article is to examine the psychological responses of college students when they are presented with moral dilemmas. We conducted our study with 213 Chinese college students.

As noted above, the Chinese government recently proposed 24 positive core socialist values that were designed to lead people to do good things and promote positive energy. We live in an era of value pluralism, where the traditional and the modern co-exist, and the foreign and the local intermix. People are inevitably influenced by negative values as well. In addition, people are sometimes dishonest and evil. It is very important to examine the effect of these ethical values and human nature on people's behaviors and psychology.

Machiavellianism is a value system that regards human nature as inherently evil, which is similar to “Thick Face, Black Heart” or *Houhei* in Chinese (Tang and Guo, 2010). Furthermore, high *Houhei* always pretend to be kind people, but they use moral rules to restrain others in order to fight for their own interests. Machiavellianism represents a different set of values within our awareness that co-exists with the ethical values that we currently advocate and practice in Chinese society, which regard human nature as inherently good. Machiavellianism includes tendencies to maximize personal interests and to use other people as tools to achieve their goals (Karkoulian et al., 2010; Demirta and Biçkes, 2014; Czibor et al., 2017). Individuals with higher levels of Machiavellianism are more likely to act contrary to ethics (Karkoulian et al., 2010; Jones and Paulhus, 2011; Elias, 2015). They may exhibit unethical behaviors such as exaggeration of their performance and causing social harm to colleagues (Greenbaum et al., 2017). Thus, based on this empirical evidence, we hypothesize: The higher an individual's level of Machiavellianism, the higher their anxiety will be when confronted with a dilemma (H1).

**Figure 1:**
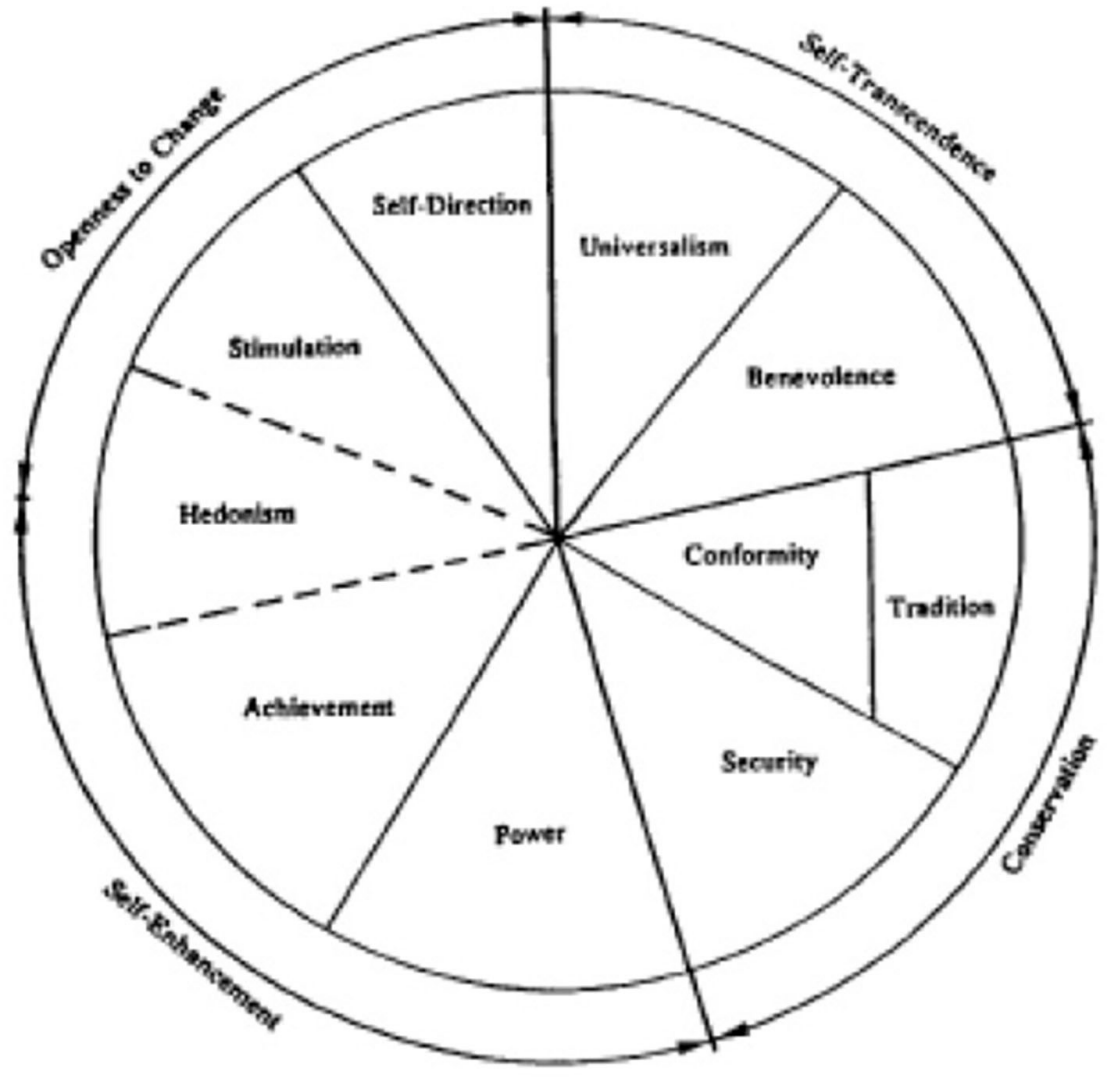
Schwartz's circumplex model of values (taken from Schwartz, 1994).

Schwartz (1994) identified 10 types of values, namely hedonism, achievement, power, stimulation, self-direction, universalism, benevolence, tradition, conformity, and security, which he placed within a circumplex model. These value types were partitioned and arranged according to two broader categories: self-enhancement vs. self-transcendence (self-directed vs. other-directed) and openness to change vs. conservation. In this model, adjacent value types are mutually complementary and harmonizing, while those situated opposite from each other have opposing and conflicting relations. Schwartz's circumplex model of values clearly depicts the structural relationships between the various types of values, as can be seen in Figure 1.

In line with this model, Machiavellianism is similar to the values of self-enhancement (self-directed values), such as the values of achievement and power, and directly opposes the values of self-transcendence (other-directed values) such as benevolence and universalism (Schwartz, 1994). Schwartz suggested that two competing or opposing values would cause psychological stress.

In order to investigate the interactive effects of Machiavellianism and ethical values on individuals' levels of psychological stress and anxiety in this study, we employed simulated situational experiments to present university students with dilemmas caused by conflicting values. Based on the theory of value conflict, we developed the following two specific research hypotheses for our study:

H2: The stronger the individual's ethical values, the higher the anxiety in the dilemma.
H3: Machiavellianism and ethical values have an interactive effect on the individual's anxiety.

## Materials and Methods

### Research Participants

Two hundred thirteen students who attended public lectures on psychology (49% male, 51% female) at a university in Hubei Province participated in this study. Participants were 20.17 years of age on average (SD = 0.79).

### Assessment Tools and Experimental Material

The following assessment tools and experimental material were used in the research:

- The 15-item Chinese Machiavellianism Personality Questionnaire compiled by Tang (2011), was used (5-point scale: 1 = *absolutely disagree*; 5 = *absolutely agree*). The total score summed across the items was divided by 15 to yield a score ranging from 1 to 5. Higher scores indicate higher levels of Machiavellian personality. The Cronbach's α coefficient was 0.79 in this study.
- The 10-item Moral Identity Scale modified by Chi (2009) was used (5-point scale: 1 = *absolutely disagree*; 5 = *absolutely agree*). Five of the items measure internalization (e.g., It's important for me to have these beautiful qualities) and five measured symbolization (e.g., I choose clothes that make me look good). The correlation between the two subscales was 0.68, so we combined them into a single scale; Cronbach's α was 0.89 in this study. Higher scores indicate higher levels of moral identity.
- The short version of the Marlowe-Crowne Social Desirability Scale (Ballard et al., 1988), which consists of 10 items, was used in this study (1 = *yes*, 0 = *no*). Higher scores indicate higher social desirability. Cronbach's α was 0.83 in this study.
- We developed five dilemmas (e.g., to maintain bitter long distance love with a girlfriend or give up and marry a more beautiful and rich girl) manifesting a conflict between Machiavellianism and moral identity (see Supplementary Material). In a pilot study, 12 psychology PhD students evaluated the dilemmas before the actual experiment began. In the Chinese cultural background, other directedness (communality) is a value that individuals are encouraged to cultivate and self-directedness (agency) is a value that is discouraged; benevolence, justice, courtesy, wisdom, and trust are also promoted. The evaluation was conducted using a 7-point scale (one indicated *completely unrelated*; seven indicated *completely related*). Each scenario was rated based on the extent to which it was related to manifestations of Machiavellianism (e.g., trying to get ahead, being unscrupulous, manipulating others, taking advantage) and to issues of moral identity (e.g., emphasizing morality and kindness, valuing others' feelings, and harmonious relationships between people). The *t*-test for paired samples was performed on the evaluation scores, and the results revealed that the scores for Machiavellianism and moral identity were not significantly different for any of the five scenarios (*M*_Mach1_ ± SD = 4.65 ± 0.36, *M*_moral1_ ± SD = 4.71 ± 0.40, *t* = 1.20, *p*_1_ = 0.32; *M*_Mach2_ ± SD = 4.34 ± 0.33, *M*_moral2_ ± SD = 4.38 ± 0.38, *t* = 0.85, *p*_2_ = 0.40; *M*_Mach3_ ± SD = 5.61 ± 0.56, *M*_moral3_ ± SD = 5.58 ± 0.53, *t* = 0.42, *p*_3_ = 0.53; *M*_Mach4_ ± SD = 4.78 ± 0.44, *M*_moral4_ ± SD = 4.84 ± 0.55, *t* = 0.91, *p*_4_ = 0.35; *M*_Mach5_ ± SD = 5.10 ± 0.46, *M*_moral5_ ± SD = 5.13 ± 0.49, *t* = 0.48, *p*_5_ = 0.63). These results indicate that all the scenarios were related to a similar extent to both Machiavellianism and ethical values.
- The seven-item Chinese version of the State Anxiety Inventory (STAI) compiled by Wang et al. (1999) was used to measure all five dilemmas. A 4-point scale was used, where one indicated “not at all,” two “somewhat,” three “moderately,” and four “very much.” The Cronbach's α was 0.90 in this study.

### Experimental Procedure

All procedures performed in this study were in accordance with the ethical standards of the Ethics Board of Central China Normal University and with the 1964 Helsinki declaration and its later amendments or comparable ethical standards. All participants provided written informed consent.

The simulated situational experiments were conducted at public psychology lectures. The first phase involved administering the Chinese Machiavellianism Personality Questionnaire, the Moral Identity Scale, and the Social Desirability Scale to the participants and informing them that the aim of filling in the questionnaires was to better understand their personality traits.

The second phase involved administering a different test packet; in this phase, participants were informed that this was part of an investigation of human relationships within the university. In the test materials administered, participants were first presented with a dilemma where Machiavellianism and ethical values were in conflict. After reading the scenario, the participants were requested to answer two questions. For the first question, “When facing such a scenario, what decision would you have made in the end?” the participants were required to choose one of two pre-set answers. For the second question, “Describe your internal feelings when you made the decision,” the participants were asked to evaluate their feelings using the STAI's 4-point scale. The first question was designed to encourage participants to think closely about the scenarios, as well as to prompt deeper reflection and processing of the presented scenarios. The participants' answers were not used in the statistical analyses. The crucial data (dependent variable) for the study were the scores from the second question (the STAI scores for each of the five dilemmas combined, so that each participant received only one STAI score).

### Statistical Analysis

The SPSS 19.0 program was used to perform statistical analyses on the collected data. We used mixed analysis of variance (ANOVA), correlation analysis, and *t*-tests in this study.

## Results

### Results of Descriptive Statistics

The top 27% were selected for the high Machiavellianism group, while the bottom 27% were selected for the low Machiavellianism group (*M*_highMach_ = 4.22, SD = 0.63, *M*_lowMach_ = 3.85, SD = 0.62, *t* = 7.22, *p* = 0.002, *d* = 0.59), comprising a total of 115 participants. Then, we divided these participants into two groups: the group with high moral identity value (which had a score higher than the median of all participants) and the group with low moral identity value (which had a score lower than the median of all participants). Thus, we obtained four groups of participants: group 1 (*n*_1_ = 36) had higher Machiavellianism and higher moral identity value, group 2 (*n*_2_ = 22) had higher Machiavellianism and lower moral identity value, group 3 (*n*_3_ = 22) had lower Machiavellianism and higher moral identity value, and group 4 (*n*_4_ = 35) had lower Machiavellianism and lower moral identity value.

The means and SDs of the STAI levels within each subgroup are shown in Table 1.

**Table 1:** Descriptive statistics for the STAI scores for the ethical dilemmas (*N* = 115).

**Machiavellianism**	**Moral identity value**	***N***	***M***	**SD**
High Machiavellianism	High moral identity value	36	13.71	2.36
	Low moral identity value	22	14.00	2.95
Low Machiavellianism	High moral identity value	22	10.91	2.14
	Low moral identity value	35	13.10	2.60

### Hypotheses Testing

The results indicated that the influence of social desirability on Machiavellianism and ethical values was not significant (*r*_Mach−desirability_ = 0.117, *p* = 0.149; *r*_ethics−desirability_ = 0.083, *p* = 0.378). Social desirability was also included as a covariate in analysis of covariance (ANCOVA) during statistical analysis, which further excluded its effects on the dependent variable.

An ANCOVA was performed to test the hypotheses. The mean STAI levels for all five dilemmas were used as the dependent variable, the levels of Machiavellianism and ethical values were used as the independent variables, and the level of social desirability was used as the covariate. The results are shown in Table 2.

**Table 2:** Analysis of covariance results.

**Variable**	**SS**	***df***	**MS**	***F***	***p***
Social desirability	12.288	1	12.288	1.948	0.166
Machiavellianism (*A*)	61.519	1	61.519	9.751	0.002
Ethical values (*B*)	29.297	1	29.297	4.644	0.033
*A* × *B*	27.002	1	27.002	4.280	0.041
Error	693.999	110	6.309		
Total	842.543	114			

**Figure 2:**
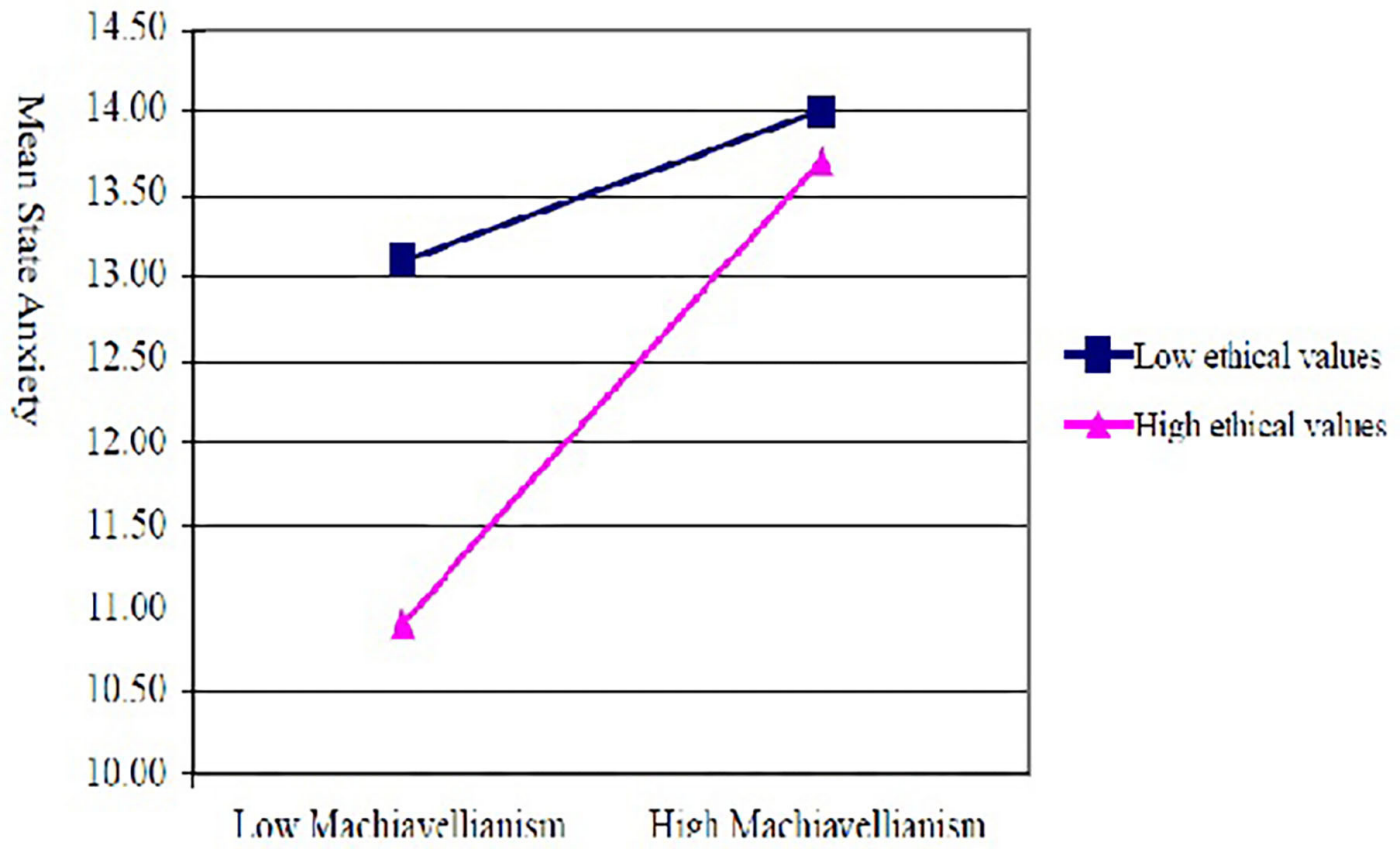
Analysis of the interactive effects between Machiavellianism and ethical values.

The findings indicated that the main effect of Machiavellianism was significant [*F*_(1,110)_ = 61.519, *p* = 0.002]. When the participants faced the dilemmas, Machiavellianism had a positive significant effect on their psychological stress or anxiety, supporting hypothesis H1. Ethical values had a significant main effect [*F*_(1,110)_ = 4.644, *p* = 0.033], demonstrating that ethical values also had a positive significant effect on participants' levels of psychological stress or anxiety when faced with ethical dilemmas, thus supporting hypothesis H2. The interaction between Machiavellianism and ethical values was also significant [*F*_(1,110)_ = 4.280, *p* = 0.041], as can be seen in Figure 2.

We further investigated the interactive effects of Machiavellianism and ethical values using ANOVAs. The results revealed that among participants with low ethical values, the STAI levels of participants with high levels of Machiavellianism (*M* = 14.00) were not significantly different from those with low levels of Machiavellianism (*M* = 13.10) [*F*_(1,111)_ = 0.82, *p* = 0.368]. However, among participants with strong ethical values, the STAI levels of those with high levels of Machiavellianism (*M* = 13.71) were significantly higher than those with low levels of Machiavellianism (*M* = 10.91) [*F*_(1,111)_ = 13.79, *p* < 0.001]. In other words, for participants with strong ethical values, their psychological stress or anxiety increased as their level of Machiavellianism increased, while participants with weaker ethical values did not experience such an effect. Therefore, this evidence supports hypothesis H3.

## Discussion

Machiavellianism is not only a personality trait, it is a value. It can affect one's relationship with others, happiness, and psychological well-being (Aghababaei and Błachnio, 2015). When faced with a dilemma involving value conflicts between Machiavellianism and ethical values, individuals will experience inner conflict, which manifests as an increase in anxiety. Ethical values also increase levels of anxiety. However, there is one other interpretation of our results: moral dilemmas are more distressing for those who do not have clear ethical values (i.e., who do not have a strong moral identity paired with low Machiavellianism). This is a very interesting hypothesis that needs to be examined in future studies.

Our findings are consistent with those of previous studies. For example, Rokeach and Ball-Rokeach (1989) suggested that if an individual's value system is in conflict, that person would experience a decrease in self-satisfaction. Burroughs and Rindfleisch (2002) believed that major conflicts arise more easily between two fundamentally opposing values, and that such conflicts will cause psychological tension in the individual, which in turn reduces their sense of well-being. Xu (2006) also reported that there is an “interdependence” between Confucianism and Machiavellianism. The correlation between Confucian concepts and impulsive tendencies was mediated by “thick black” concepts and behaviors, while the correlation between Machiavellianism and impulsive tendencies was mediated by Confucian concepts and behaviors (Xu, 2006).

Values are gradually established over an individual's lifetime as he or she accumulates knowledge and life experiences, but once the individual's values have been established, they provide a certain level of stability, producing set value orientations and behaviors that are difficult to change. Evidently, when an individual possesses both Machiavellianism and ethical values simultaneously, for a time he or she will hold conflicting values before a stable value system is established and re-prioritization occurs, and therefore internal conflicts are inevitable.

There are some caveats that should be noted in the present study. First, because of the correlational nature of these findings, causal interpretations are not appropriate. Second, as the sample is not representative of the entire population, future studies should test this model with a larger and gender and age heterogeneous sample.

## Conclusion

When participants were faced with dilemmas, Machiavellianism had a significant effect on their psychological stress or anxiety. The higher the individual's level of Machiavellianism, the higher his or her level of psychological stress or anxiety. Ethical values also had a significant effect on the psychological stress or anxiety they experienced when confronted with a dilemma. The stronger an individual's ethical values, the higher their level of psychological stress or anxiety.

Machiavellianism and ethical values had an interactive effect on individuals' levels of psychological stress or anxiety. Among individuals with high levels of ethical values, their psychological stress or anxiety increased as their level of Machiavellianism increased. However, among individuals with low levels of ethical values, there were no differences in psychological stress and anxiety between those with low and high levels of Machiavellianism.

## Data Availability Statement

The raw data supporting the conclusions of this article will be made available by the authors, without undue reservation.

## Ethics Statement

The studies involving human participants were reviewed and approved by the Ethics Board of Central China Normal University. The patients/participants provided their written informed consent to participate in this study.

## Author Contributions

ST: developed and validated the theoretical model, data preparation and analysis, and manuscript writing. KL: expanded the theoretical model and manuscript writing. Both authors contributed to the article and approved the submitted version.

## Conflict of Interest

The authors declare that the research was conducted in the absence of any commercial or financial relationships that could be construed as a potential conflict of interest.
