# Supplementary material for: The Influence of Chinese Machiavellianism and Moral Identity on the Level of Anxiety in Moral Dilemma Situations in Chinese Students
Source: Front Psychol. 2021 Feb 25;12:615835. doi: 10.3389/fpsyg.2021.615835 (PMC7947343; doi:10.3389/fpsyg.2021.615835)
Supplement: Supplementary file 1 [file Data_Sheet_1.pdf]

Supplementary Material

Scenarios 1: Suppose you have been in love with your girlfriend for many years, but you work in different places, so emotional communication is inconvenient. Your colleague introduced you to a girl with a very good family financial situation. You are more tempted. But your girlfriend intends to resign and go to the city where you work to continue the relationship. Will you use this new relationship to force your girlfriend to break up?

Please answer:

1. In the face of this situation, what decision will you make in the end?

A. Force his girlfriend to break up B. Renew the relationship with his girlfriend

2. The following statements are used to describe your feelings during the decision-making process. Please determine your best according to your own feeling. Please judge each item according to the scale method of 1-4 points below, and tick "√" on the corresponding number.

1 I feel calm 1 2 3 4

2 I feel nervous 1 2 3 4

3 I feel upset 1 2 3 4

4 I am dissatisfied 1 2 3 4

5 I am indecisive 1 2 3 4

6 I feel uncomfortable 1 2 3 4

7 I feel conflicted 1 2 3 4

Scenarios 2: Suppose you are a fresh graduate of your senior year, and a business unit has appointed a new employee to your class. You are very interested in this. Several other students in your class have also submitted resumes. Among them, Li's academic performance and school league experience are your biggest competitors. It happens that the counselor teacher arranged for you to send all your resumes to the company. Will you take the opportunity to take out Li's resume?

Please answer:

1. In the face of this situation, what decision will you make in the end?

A. take out Li's resume B not take out

2. The following statements are used to describe your feelings during the decision-making process. Please determine your best according to your own feeling. Please judge each item according to the scale method of 1-4 points below, and tick "√" on the corresponding number.

1 I feel calm 1 2 3 4

2 I feel nervous 1 2 3 4

3 I feel upset 1 2 3 4

4 I am dissatisfied 1 2 3 4

5 I am indecisive 1 2 3 4

6 I feel uncomfortable 1 2 3 4

7 I feel conflicted 1 2 3 4

Scenario 3: Suppose you were caught on the spot for cheating on a mobile phone in a CET-4 test. The teacher in the Office of Academic Affairs wants you to confess all the circumstances, and one of your classmates assisting you in cheating. In your school, cheating in the Level 4 exam will result in expulsion of students. The dean encourages you to report and expose, and strive for leniency. Will you confess your classmate?

1. In the face of this situation, what decision will you make in the end?

A. confess my classmate B not confess my classmate

2. The following statements are used to describe your feelings during the decision-making process. Please determine your best according to your own feeling. Please judge each item according to the scale method of 1-4 points below, and tick "√" on the corresponding number.

1 I feel calm 1 2 3 4

2 I feel nervous 1 2 3 4

3 I feel upset 1 2 3 4

4 I am dissatisfied 1 2 3 4

5 I am indecisive 1 2 3 4

6 I feel uncomfortable 1 2 3 4

7 I feel conflicted 1 2 3 4

Scenario 4: Your college is currently running for the president of the student union. You and Wang are both popular candidates for the position, and each has advantages and is comparable. You have always been in close contact with Zhang, but Zhang has gradually become at odds because he lives in

the same dormitory with Wang. During the critical period of the competition for employment assessment, would you encourage Zhang to report some negative news about Wang to the counselor?

1. In the face of this situation, what decision will you make in the end?

A. encourage Zhang to report some negative news about Wang B. not

2. The following statements are used to describe your feelings during the decision-making process. Please determine your best according to your own feeling. Please judge each item according to the scale method of 1-4 points below, and tick "√" on the corresponding number.

1 I feel calm 1 2 3 4

2 I feel nervous 1 2 3 4

3 I feel upset 1 2 3 4

4 I am dissatisfied 1 2 3 4

5 I am indecisive 1 2 3 4

6 I feel uncomfortable 1 2 3 4

7 I feel conflicted 1 2 3 4

Scenario 5: Your college is now in the selection of the annual "Three Good Students". You and the poor student Zhao in your class are both nominated. The title of "Three Good Students" is a necessary condition for graduate school. You want to continue to study in this school, so you are very This selection is very important. Zhao also attaches great importance to it, and at the same time, you understand that Zhao has no intention of going to graduate school. Would you take the initiative to find Zhao to negotiate with him to withdraw from the three-good students and give him some financial compensation?

Please answer:

1. In the face of this situation, what decision will you make in the end?

A. to negotiate with him to withdraw from the three-good students and give him some financial compensation B. not

2. The following statements are used to describe your feelings during the decision-making process. Please determine your best according to your own feeling. Please judge each item according to the scale method of 1-4 points below, and tick "√" on the corresponding number.

1 I feel calm 1 2 3 4

2 I feel nervous 1 2 3 4

3 I feel upset 1 2 3 4

4 I am dissatisfied 1 2 3 4

5 I am indecisive 1 2 3 4

6 I feel uncomfortable 1 2 3 4

7 I feel conflicted 1 2 3 4
